# Gender Equality and Health in High-Income Countries: A Systematic Review of Within-Country Indicators of Gender Equality in Relation to Health Outcomes

**DOI:** 10.1089/whr.2020.0114

**Published:** 2021-04-27

**Authors:** Allison Milner, Anne Kavanagh, Anna J. Scovelle, Adrienne O'Neil, Guyonne Kalb, Belinda Hewitt, Tania L. King

**Affiliations:** ^1^ Centre for Health Equity, School of Population and Global Health, University of Melbourne, Melbourne, Australia.; ^2^ Deakin University, IMPACT–The Institute for Mental and Physical Health and Clinical Translation, School of Medicine, Barwon Health, Geelong, Australia.; ^3^ Centre for Mental Health, Melbourne School of Population and Global Health, University of Melbourne, Melbourne, Australia.; ^4^ Melbourne Institute of Applied Economic and Social Research, University of Melbourne, Melbourne, Australia.; ^5^ School of Social and Political Sciences, University of Melbourne, Melbourne, Australia.

**Keywords:** gender equality, health, review

## Abstract

***Background:*** Gender equality is recognized as an important political, social, and economic goal in many countries around the world. At a country level, there is evidence that gender equality may have an important influence on health. Historically gender equality has mainly been measured to allow for between-country, rather than within-country comparisons; and the association between gender equality and health outcomes within countries has been under-researched. This article thus aimed to systematically review within-country indicators of gender equality in public health studies and assess the extent to which these are related to health outcomes.

***Materials and Methods:*** We used the Preferred Reporting Items for Systematic Reviews and Meta-Analyses (PRISMA) approach with two independent reviewers.

***Results:*** Data from the eight included studies revealed that there was heterogeneity in the way gender equality has been measured as a multidimensional construct. Associations between gender equality and a number of different health outcomes were apparent, including mortality, mental health, morbidity, alcohol consumption, and intimate partner violence, with gender equality mostly associated with better health outcomes.

***Conclusions:*** Further investigation into the effects of gender equality on health outcomes, including a clear conceptualization of terms, is critical for the development of policies and programs regarding gender equality.

## Introduction

The World Health Organization (WHO) recognizes gender as a key driver of inequalities in living conditions and, by extension, health.^1^ “Gender equality” refers to the entitlement of all genders to enjoy equal rights, opportunities, and treatment. Acknowledging that men and women are not the same, gender equality asserts that all genders have the right to develop and pursue their interests free of discrimination, stereotypes, and biases.^2^ Progressing gender equality is recognized as an important political, social, and economic goal in many countries around the world.^3,4^ Moreover, there is evidence that gender equality has an important influence on health,^5^ and indeed some claim that gender inequality represents one of the most significant threats to global health.^6^

The social determinants of health (SDOH) framework recognizes the conditions in which people are born, grow, work, live, and age, as well as the broader forces and systems that shape these conditions, such as social policies, social norms, and political systems,^7^ and has previously been applied to studies of gender equality.^5,8^ According to the SDOH framework, gender inequalities are socially generated, and influence health through a multitude of mechanisms, including violence, lack of power, lack of resources, and inequitable divisions of work and leisure.^1^

Heise et al.^9^ have drawn on the life-course perspective^10^ and the SDOH framework^7^ to propose a conceptual framework by which gender, gender norms, and gender inequality impart effects on health, arguing that individuals are embedded in the gender system across the life course, and across many life domains. These domains include family, community, institutions, and structures such as education, the workforce, and political institutions, as well as policies. It is across and through these that norms are imbibed, enacted, reinforced, and enforced, and power is experienced and distributed.^9^

The extent to which these important conceptual understandings are captured in research that measures the impact of gender equality on health is questionable. Indeed, measurement of gender equality is essential to understand and monitor progress of this process and to determine its impact on health. To this end, a number of gender equality indices and tools have been developed. Prominent examples include the Gender Inequality Index (GII), the Gender Empowerment Measure (GEM), the Gender Development Index (GDI) (all produced by the United Nations Development program^11^), and the Global Gender Gap Index (GGGI) (produced by the World Economic Forum).^12^ These indicators summarize gender equality across a number of domains, including economic participation and opportunity, education attainment, health and survival, and political empowerment. However, these measures utilize data aggregated at the country level, and have been primarily developed to facilitate international comparisons. As such, their composition is often (at least partly) driven by the availability of data that can facilitate comparison across a large group of countries, rather than being conceptually driven. Furthermore, many of these indicators are most appropriate for comparison across low- and middle-income settings.^13,14^ Gender equality varies substantially across low- and middle-income countries, but is typically lower in these contexts compared with high-income countries. This is attributed to differences in the overall levels of education, distribution of resources and wealth, as well as strengths of the health system.^15^ Given this, the utility of such measures when examining and assessing gender equality in high-income countries is limited and may mask important gender inequalities within those countries. In addition, such indicators do not capture the multiple domains across which gender equality operates (*e.g.*, family, workplace, community).

Despite the fact that many high-income countries have made substantial gains in many elements of gender equality such as education, gender inequalities persist across many domains such as employment, wages, and political representation,^16^ as well as health and health systems.^15^ Assessing and monitoring gender equality within high-income countries therefore remain a priority. While there is a clear need to understand how to assess and monitor gender equality in high-income countries, there is a paucity of evidence regarding how gender equality should be measured in these contexts, particularly in relation to health outcomes. Furthermore, the association between gender equality and health in high-income countries is poorly understood. Given the impetus underscored by the aforementioned points, the objectives of this article were first, to systematically review within-country multidimensional indicators of gender equality that have been used as exposures in public health studies in high-income countries, and second, to assess the extent to which these are related to health outcomes.

## Methods

We conducted the search according to the Preferred Reporting Items for Systematic Reviews and Meta-Analyses (PRISMA) guidelines.^17^

### Search strategy

The peer-reviewed literature of four databases was searched: PubMed, Global Health, PsycInfo, and Scopus in March 2017. No restrictions were placed on publication date, language, or publication type. The search terms were based on a three-tiered search strategy. The first tier represented terms related to gender equality (“gender equality,” “gender equity,” “gender inequality,” “gender inequity,” “women”). The second tier represented health outcomes (“health,” “morbidity,” “mortality,” “health behaviours,” “mental health”), and the last tier represented research design (“ecological,” “panel study,” “longitudinal design,” “observational,” “cohort”). Each of these search tiers were run separately, and then all three tiers were combined into one total search.

### Study selection and inclusion criteria

Two authors (A.M. and T.L.K.) independently screened articles. We sought empirical studies that examined associations between gender equality indicators and health outcomes in high-income countries. As gender equality is a complex and multidimensional concept, we sought studies that used multidimensional measures of gender equality. For inclusion in the review, each study must have:

1. been conducted within a country categorized as high income, as defined by the World Bank^18^;
2. used a multidimensional measure of gender equity/inequity/equality/inequality as the exposure;
3. included “gender equity/inequity/equality/inequality” in the title; and
4. used a measure of health as the outcome (as measured by self-report, doctor diagnosis, medical records, health administration records).

We excluded studies:

1. of qualitative design;
2. that were commentaries, purely theoretical or descriptive analysis that did not examine associations (given our focus on measured associations between gender equality and health);
3. that were conducted in low- or middle-income or developing countries;
4. that assessed gender equality as a single dimension; and
5. that used common global measures of gender equality. As these global measures were developed to compare gender equality across countries, they rely on data aggregated at the country level. We sought measures of gender equality that had been developed to compare gender equality within countries.
6. that did not explicitly state that their key exposure was gender equity/inequity/equality/inequality.

Where consensus on these criteria was not reached by the two reviewing authors, a third author (A.K.) was consulted.

### Data extraction

Two reviewers (A.M. and T.L.K.) extracted data using a standardized template across the following categories: country/study population, study aim, study design, outcome, measure of gender equality, and results (Table 1). All discrepancies were resolved through discussion between team members.

**Table 1.** Key Characteristics of the Studies Included in this Article

Author, year	Country, area level, study population	Aim	Design	Measurement of gender equality	Outcome	Results
Backhans et al. 2007	Sweden Population statistics from *N* = 289 Swedish municipalities	To test the hypothesis that greater gender equality is associated with better health outcomes	Cross-sectional study/linked population data	289 Swedish municipalities • Political participation: proportion of women versus men in municipal councils and executive committees • Division of labor (public sphere): (1) temporary parental leave (proportion taken by men vs. women); (2) proportion of part-time workers • division of labor (private sphere): (1) proportion of men and women in health care and social services; (2) proportion of men and women in manufacturing; (3) proportion of men and women in managerial positions • Economic resources: average income, relative poverty	Linked individual data: • life expectancy • number compensated days per insured person for sickness absence and disability (men and women)	Gender equality associated with higher levels of sickness and disability for men and women. Gender equality associated with lower life expectancy.
Chen et al. 2005	The United States (50 U.S. States) *N* = 7,789 women in a 1991 longitudinal study (participants were a nationally representative random sample of women who gave birth to live babies in 1988)	To assess the relationship between state-level women's status variables and individual depressive symptoms	Follow-up study of participants of a nationally representative sample: multilevel analysis of individuals nested in states	U.S. state level: • political participation • employment and earnings • social/economic autonomy • reproductive rights	Individual-level data: symptoms of depression (Centre for Epidemiological Studies-Depression) (women)	Women residing in states with high scores of employment and earnings on index had lower depression than those who scored lower on the index. Women in states with higher scores on economic autonomy had lower depression. Women in states with high reproductive rights had lower depression.
Jun et al. 2004	The United States *N* = 87,848 women in the Behavioral Risk Factor Surveillance System study	Examine associations between self-rated health and women's status	Cross-sectional: multilevel analysis of individuals nested in states	Women's status at the state level: • political participation • economic autonomy • employment and earnings	Individual-level data: Self-rated health	Low status for women was associated with higher likelihood of reporting poor health.
Kawachi et al. 1999	The United States (50 U.S. States) Age-standardized, cause-specific, mortality statistics	Examine associations between the status of women, and women and men's health status	Ecological and cross-sectional	Women's status at the state level: • political participation • employment and earnings • social/economic autonomy • reproductive rights	State-level data: • total female and male mortality rates female cause-specific death rates • Mean days of activity limitations reported by women in the previous month	Higher political participation by women correlated with lower female mortality rates and lower activity limitations. Smaller wage gap associated with lower female mortality rates and lower activity limitations. Indices of women's status were correlated with male mortality rates; such that higher mortality rates were associated with lower status of women. Associations between indices of women's status and female mortality rates persisted after adjustment for income inequality, poverty rates and median household income.
McLaughlin et al. 2011	The United States (50 U.S. States) National probability sample of U.S. adults (*n* = 34,653); data from the National Epidemiological Survey on Alcohol and Related Conditions	To what extent is state-level women's status related to psychiatric disorders in women and gender differences in psychopathology?	Cross-sectional survey	Women's status at the state level: • political participation • employment and earnings • social/economic autonomy • reproductive rights	Individual-level data: 12-month mood and anxiety disorders (women)	The prevalence of major depression and post-traumatic stress disorder (PTSD) was lower in states where women have reproductive rights. Other variables of state-level women's right variables were unrelated to depression and anxiety.
Roberts 2012	The United States (50 U.S. States) Survey data from the 2005 Behavioral Risk Factor Surveillance System study	To understand the relationship between state-level gender equality and alcohol consumption	Cross-sectional survey: multilevel analysis (hierarchical linear modeling) of individuals nested in states	State level • women's socioeconomic status (absolute) • gender equality in socioeconomic status (relative) • reproductive rights • policies relating to violence against women • women's political participation	Individual-level data: alcohol consumption including past 30-day drinker status, drinking frequency, binge drinking, volume, risky drinking (men and women)	Greater gender equality on all indicators was associated with less alcohol consumption in women and men. Findings do not support the hypothesis that high gender equality on women's status is associated with higher alcohol consumption.
Yllo 1983	The United States (30 U.S. States) Individuals cohabiting	Investigate association between women's status and IPV	Ecological and cross-sectional	Dimensions of Status of Women's index: economic, educational, political, and legal dimensions.	State-level data: proportion of couples who reported that husband had used violence against wife	A curvilinear relationship exists such that in states where the status of women is low, IPV rates against women are high. IPV rates decline with increasing status of women; however, when the status of women is at its highest, IPV rates are once again high.
Yllo 1984	The United States (30 U.S. States) Individuals cohabiting	Investigate whether sexual inequality is associated with the relationship between marital inequality and IPV	Cross-sectional	Dimensions of Status of Women's index: economic, educational, political, and legal dimensions.	State-level data: proportion of couples who reported that husband had used violence against wife	Higher levels of IPV in couples where husband dominates decision making, residing in states in which there is high status for women (high gender equity). Also, higher levels of IPV in couples where wife dominates decision making, residing in states with low status for women (low gender equity).

IPV, intimate partner violence.

## Results

### Search strategy

**FIG. 1.**
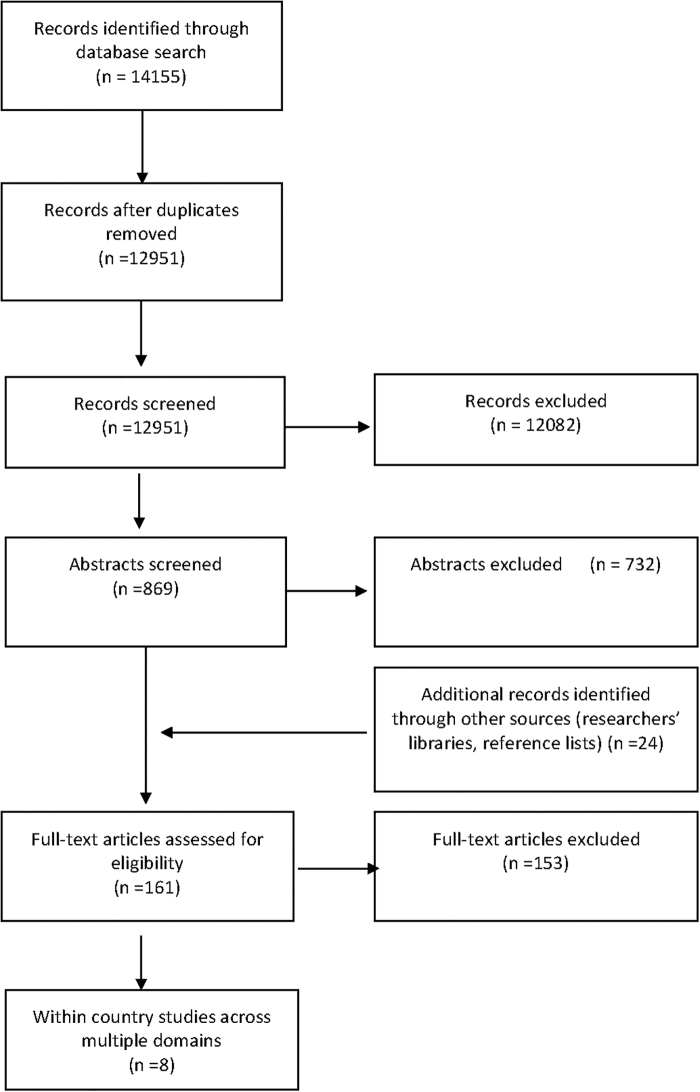
Flow diagram of study selection.

Figure 1 summarizes the sample selection process. Initially, 14,155 peer-reviewed journal publications were identified through the database search. After the removal of 1,204 duplicates, 12,951 publications remained for consideration. All publication titles were first screened, and titles were excluded when it was apparent that inclusion criterion was not met. At this stage, 12,082 publications were excluded for reasons including that they (1) were not conducted in a high-income country; (2) did not contain “gender equity/inequity/equality/inequality” in the title; (3) did not contain health or a measure of health in the title; or (4) were not research articles (*i.e.*, were commentaries or theoretical publications). The abstracts of the remaining 869 studies were screened. Of these, 732 were excluded for not meeting the inclusion criteria. At this stage, a further 24 records were identified through reference lists of eligible studies and authors' libraries. After full-text review of the remaining 161 articles, 8 studies meeting the inclusion criteria were retained.

Key characteristics of included studies are summarized in Table 1. These studies covered a range of time periods and different outcomes. Notably, the overwhelming majority (seven) of studies were conducted in the United States, with one study in Sweden.

### Gender equality measures and their relationship to health outcomes

The section below provides a description of how gender equality was measured in each of the eight publications included in the review. We also briefly present the findings for the relationship between gender equality and health outcomes, including mortality, mental health, morbidity, alcohol consumption, and intimate partner violence (IPV). A more detailed review of the relationship between gender equality and health has been recently published by the authors.^19^

The indicators of gender equality featured in the reviewed studies were operationalized at the state level for all seven U.S. studies and the municipality level for the Swedish study. Four studies in the United States^20–23^ used a measure of the status of women that was developed and published by the Institute for Women's Policy Research.^24^ They created four separate composite indices across four domains: (1) political participation/representation, (2) social/economic autonomy, (3) employment/earnings, and (4) reproductive rights. Political participation includes four absolute indicators: percentage of women registered to vote, percentage of women who voted, representation in elected office, and existence of institutional resources for women. Four absolute measures constitute the social/economic autonomy indicator: proportion of women with health insurance, women's educational attainment, women's business ownership, and proportion of women living above the federal poverty level. Employment and earnings also include four indicators that are a mix of both relative and absolute measures: women's median annual earnings, ratio of women's to men's earnings, women's labor force participation, and women's representation in managerial and professional occupations. Each study used slightly different absolute reproductive rights indicators, including a combination of mandatory parental consent/notification abortion laws for minors, mandatory abortion waiting period, public funding for abortion, presence of prochoice legislature or governor, proportion of women living in areas with at least one abortion provider, mandatory sexual education, contraceptive coverage laws, coverage of infertility treatments, and legality of same-sex couple adoption.

Among these studies, Kawachi et al.^22^ found that higher female political participation and smaller gender wage gaps were associated with reduced mortality rates in women and men, and less disability in women. Chen et al.^21^ reported that women who lived in states with higher levels of gender equality in employment earnings, economic autonomy, and reproductive rights had lower levels of depression. Similarly, McLaughin et al.^20^ demonstrated that the prevalence of major depression and post-traumatic stress disorder (PTSD) was lower in states where women had more reproductive rights. Jun et al.^23^ found that in states with lower levels of gender equality, women were more likely to report poor self-rated health.

Another study in the United States by Roberts^25^ incorporated similar measures of reproductive rights and political participation as described above, but also included absolute and relative measures of socioeconomic status (state-level women's socioeconomic status and gender equality in social-economic status, respectively), and policies to combat violence against women. This study found that greater gender equality was associated with lower alcohol consumption for both men and women.

Two papers by Yllo^26,27^ in the United States assessed gender equality across four separate dimensions (these were not combined) that used a combination of relative and absolute measures. The dimensions were as follows: economic (which included median income, but was principally operationalized in terms of labor force participation with indicators such as the percentage of women in the labor force and the male unemployment rate as a percentage of the female rate), education (including four indicators, such as female high school graduation rate as a percentage of the male rate and percentage of female postsecondary enrollment), political representation (including four indicators, such as the percentage of female members in congress and the percentage of members of the state senate), and legal (including 14 indicators, such as no occupations barred to women, fair employment practices act, and proof of resistance not required for rape conviction). A curvilinear relationship was revealed, such that in states where gender equality was low, IPV rates were high. IPV rates declined with increasing status of women; however, when the status of women was at its highest, IPV rates were once again high.^26^ Interestingly, Yllo^27^ later reported that couples where the husband dominated decision making (self-reported) and who lived in areas with greater gender equality had higher levels of IPV. Similarly, couples where the wife dominated decision making and who lived in areas of lower gender equality also had higher levels of IPV.^27^

For the Swedish study, the researchers measured gender equality^28^ using a combination of indicators across three broad dimensions: political participation (proportion of women vs. men in municipal councils and municipal executive committees); division of labor in the private sphere (proportion of temporary parental leave taken by women vs. men, proportion of part-time workers in women and men), and public sphere (proportion of men vs. women for people employed in female- vs. male-dominated occupations; proportion of women vs. men in managerial positions); and economic resources (average income and relative poverty for females and males). Using the derived relative measure, they reported that greater gender equality was associated with higher levels of sickness and disability, and reduced life expectancy for both women and men.

Our systematic analysis of these studies conducted in high-income countries revealed seven interconnected domains for which gender equality was conceptualized and measured. These were labor force participation; political and public representation; economic resources/income; division of labor within the home; leave and entitlements; rights (*e.g.*, health, reproductive, economic); and education (see Table 2 for a summary). We note that no study included all of these domains, and division of labor within the home was only measured in one study.

**Table 2.** The Domains of Gender Equality in Each Paper Reviewed

	Labor force participation	Political and public representation	Economic resources/income	Division of labor within the home	Leave and entitlements	Rights (health and reproductive, legal)	Education
Backhans et al. 2007	✓	✓	✓	✓	✓		
Chen et al. 2005	✓	✓	✓			✓	✓
Jun et al. 2004	✓	✓	✓			✓	✓
Kawachi et al. 1999	✓	✓	✓			✓	✓
McLaughlin et al. 2011	✓	✓	✓			✓	✓
Roberts 2012		✓	✓			✓	
Yllo et al. 1983	✓	✓	✓			✓	✓
Yllo et al. 1984	✓	✓	✓			✓	✓

## Discussion

This article systematically reviewed studies that examined within-country indicators of gender equality in relation to health. Data from the eight included studies revealed that gender equality has been measured as a multidimensional process embedded in a variety of domains. Associations between gender equality and a number of different health outcomes were shown. Below, we first discuss the associations between gender equality and health measures that arose in the included studies; we discuss the strengths and weaknesses of different measures of gender equality; and we then suggest ways in which the measurement of gender equality can be strengthened in public health research.

### Associations between gender equality and health

A variety of health outcomes were assessed in relation to gender equality, with the majority being related to mental health. In almost all studies, higher levels of gender equality were associated with better health outcomes, including reduced depression and PTSD, reduced mortality rates, better self-rated health, and reduced alcohol consumption. The one non-U.S. study, from Sweden,^28^ found that higher gender equality was associated with poorer health outcomes, including sickness and disability, and reduced life expectancy. While Yllo^26^ found that on average higher levels of gender equality were associated with lower levels of domestic violence, this relationship was curvilinear where the states ranked in the bottom and top 20% for gender equality had the highest rates of domestic violence and it was also dependent on decision making within the couple relationship.^27^ On balance, however, the findings of this review align with other research, indicating that on the whole, gender equality is associated with better health outcomes.^19^

### Gender equality measures

Seven domains of gender equality were identified or measured across the eight studies included in this review. These domains map onto the WHO definition of the social structures that influence health and broadly align with the SDOH.^29^ Dimensions such as education, labor force participation, and political representation that were used in the included studies align with those included in some of the established intercountry measures such as the GGGI and the GII. However, the division of household labor or leisure time, identified as a key driver of gender inequalities in health according to the SDOH framework,^1^ is a critical omission of indicators such as the GGGI and GII, and an important inclusion in the Swedish study included in this review.^28^ Another key difference between the measures included here and the country comparative measures such as the GGGI and GII is in relation to maternal mortality and adolescent fertility rates—no such measures were included in the studies in this review, reflecting the fact that maternal mortality and adolescent fertility are both extremely low in most high-income countries.^16^

A significant strength of gender equality measurement in the studies in our review was that studies recognized gender equality to be fundamentally underpinned by the concept of social status. Others have noted that scholarship examining gender equality over the past 30 years has shared the objective to examine the differential social statuses of women and men.^30^ This includes seeking to quantify the economic resources that women share relative to men, their positions of power, and their legal rights. Another important strength of the indicators included was that they were operationalized at the state/municipal level, rather than country level (like many of the more well-known measures such as the GDI, GII, GGGI, or GEM). The fact that the studies were conducted within countries also has the benefit of controlling for many contextual factors associated with legal and health systems, social norms, and educational and other institutions (acknowledging some variations associated with governance and distribution of power within countries— *e.g*., variations in access to abortion services). What is notable is that while studies conceptualized gender equality as being a multidimensional concept, they typically examined it across the separate dimensions, rather than as an overall composite measure.

A weakness of all but one study reviewed is that they omitted gender equality in terms of caring and domestic work. That is, they did not assess the relative contribution of women and men within the domestic sphere to either housework or the care of dependents. There is growing recognition that time spent on domestic tasks is an important resource and underpins gender equality.^31^ In addition, most of these studies would have benefited from evaluating gender equality in a broader range of structural dimensions, as discussed in the next section. A further limitation is that some gender equality indicators contained health measures. This is not problematic in itself, but there are substantial methodological implications when the indicator is assessed in relation to a health outcome, as it may lead to conflation between the exposure and outcome. Importantly too, several of the indicators combined relative and absolute measures. The combination of relative measures (indicators of women's achievement/status relative to men) with absolute measures of women's achievement has been subject to scholarly criticism, as the two are considered to be conceptually incompatible.^14,30^

### Implications of review findings for the measurement of gender equality in high-income countries

This review has highlighted the dearth of studies that have utilized multidimensional measures of gender equality to examine associations between gender equality and health outcomes within high-income countries. This represents an important avenue for future research. Importantly, there is a clear need for conceptual clarity regarding the components of gender equality measures when applied to health outcomes. Many of the studies included here drew on a measure of gender equality that was developed by the Institute for Women's Policy Research.^24^ While seminal, this was intended as a tool for the assessment of gender equality broadly. The development and application of a tool that has conceptual clarity and pertinence to health outcomes is needed. Some of the measures incorporated into the included studies provide a useful starting point. In particular, it is clearly important that gender equality measures capture equality in terms of labor force participation, equal representation across different occupations and industries, and representation in different leadership positions (*e.g.*, proportion of women in managerial positions). To this, we would also add the need to consider equality in forms of employment arrangements (*e.g.*, equal access to part-time and full-time working arrangements). Representation and leadership by male and female politicians at various levels of government are also important. This covers a range of political positions within a country across bodies both in and out of government, as well as the equal recognition of men and women in fields of excellence (*e.g.*, science, business, or community service).

Gender equality measures also highlight the importance of equal access to income and financial capital, as well as overall wealth, recognizing that women on average have a shorter working life (often at lower pay) than men due to child rearing, and therefore have accumulated fewer individual savings and assets for retirement.^32^ The division of labor within the home is also an important component that many existing measures do not include, with only one study in this review including a measure of the division of household labor.^28^ As noted, this is an important omission, as the way that household labor is divided in households directly underpins women's capacity to enter employment and maintain workforce attachment and as such, is one of the most persistent determinants of gender equality, particularly in high-income countries. Indicators in this domain could measure the extent to which men and women take responsibility for the care of family. This should include time taken for the care of children, care for older parents and relatives, and care for others within the community. Alongside this, measurement of gender equality should acknowledge the differences in contributions to the household; for example, the time taken to organize household finances and to contribute to household chores. Related to this, some measure of unpaid time use/leisure time is also important.^13^

Leave and entitlements refer to the extent to which men and women have access to parental leave and social welfare/income protection, and should also be integral to gender equality measures. In many developed countries, participation in education has reached gender parity, so educational measures must extend beyond participation/enrollment and literacy, and include level of educational attainment, participation in higher education, and level of educational segregation (whether women and men select different types of studies).^30^

### Limitations of the review

In terms of limitations, we acknowledge that the scope of this article has been confined to research in public health. We are aware that the issues regarding the conceptualization and measurement of gender equality have been discussed across a number of different domains,^13,14,30,33^ including sociology, anthropology, and economics, and a number of diverse theories have been applied. Further, the review is specific to quantitative within-country studies; however, we would encourage qualitative research and other designs to pursue evaluations of associations between gender equality and health. Importantly too, our discussion of gender equality has focused on high-income contexts rather than low- and middle-income settings, so the extent to which the findings can be generalized to settings other than high-income countries is not known. Further, the domains we present as constituting gender equality are those that are amenable to quantitative measurement.

We also acknowledge that while gender is not binary,^34^ the literature included in this review takes a binary approach and only examines equality between men and women. As a further point, we argue that public health researchers should examine gender equality as a separate construct to the broader construct of gender. Gender is an important social determinant of health,^35,36^ and is defined by norms, roles, and relationships within and between groups of women and men.^37^ Gender-based differences in health should be distinguished from the impact of gender equality on health, given that gender equality denotes structures and access to resources.

Some further limitations are related to the inclusion and exclusion criteria. First, we sought studies that had used multidimensional measures of gender equality, and in doing so excluded studies using single measures of gender equality. It is also possible that some relevant studies have been omitted from this review because their title did not contain “gender equity” or “gender equality.” An additional limitation is that the literature search for this review was conducted in 2017, and it is likely that additional in-scope studies have been published since then.^38^ We also note that as we were focused on studies that had examined gender equality *within* countries, we excluded studies that utilized global measures of gender equality. This approach was intended to capture those studies that examined gender equality at a granular level (rather than between large jurisdictions). However, it still does not fully capture the multiplicity of experiences of gender equality, nor the way in which these experiences may vary across different intersections of disadvantages such as race and class.

## Conclusion

In conclusion, this review has identified a small number of studies that have applied multidimensional measures of gender equality in relation to health outcomes in high-income countries. For almost all of the included studies, gender equality was found to have a positive association with the different health outcomes assessed. This review has highlighted that understanding the relationship between gender equality and health requires clear conceptualization and measurement of gender equality itself. Measures that are designed to capture across national differences may not adequately capture gender inequality in high-income countries. Information from further in-depth investigation into the effects of gender equality on health outcomes is critical for the development of policies and programs regarding gender equality.

## Abbreviations Used

GDI: Gender Development Index
GEM: Gender Empowerment Measure
GGGI: Global Gender Gap Index
GII: Gender Inequality Index
IPV: intimate partner violence
PTSD: post-traumatic stress disorder
SDOH: social determinants of health
WHO: World Health Organization
